# Changes in Mitochondrial Function and Cell Death Patterns in Peripheral Blood Mononuclear Cells during Trastuzumab Treatment Following Doxorubicin Chemotherapy

**DOI:** 10.3390/biomedicines12091970

**Published:** 2024-09-01

**Authors:** Krit Leemasawat, Nichanan Osataphan, Nattayaporn Apaijai, Panat Yanpiset, Arintaya Phrommintikul, Areewan Somwangprasert, Siriporn C. Chattipakorn, Nipon Chattipakorn

**Affiliations:** 1 Cardiology Division, Department of Internal Medicine, Faculty of Medicine, Chiang Mai University, Chiang Mai 50200, Thailand; krit_lee@yahoo.com (K.L.); aom.osataphan@gmail.com (N.O.); arintayap@yahoo.com (A.P.); 2 Cardiac Electrophysiology Research and Training Center, Faculty of Medicine, Chiang Mai University, Chiang Mai 50200, Thailand; napaijai@gmail.com (N.A.); panatbonus@gmail.com (P.Y.); scchattipakorn@gmail.com (S.C.C.); 3 Center of Excellence in Cardiac Electrophysiology Research, Chiang Mai University, Chiang Mai 50200, Thailand; 4 Department of Surgery, Faculty of Medicine, Chiang Mai University, Chiang Mai 50200, Thailand; asomwang@yahoo.com; 5 Department of Oral Biology and Diagnostic Sciences, Faculty of Dentistry, Chiang Mai University, Chiang Mai 50200, Thailand; 6 Cardiac Electrophysiology Unit, Department of Physiology, Faculty of Medicine, Chiang Mai University, Chiang Mai 50200, Thailand

**Keywords:** cardiotoxicity, cell death, mitochondria, oxidative stress, peripheral blood mononuclear cell, trastuzumab

## Abstract

Trastuzumab, a monoclonal antibody which works against human epidermal growth factor receptor 2 (HER2), possibly causes cardiotoxicity through mitochondrial dysfunction. The usefulness of isolated peripheral blood mononuclear cells (PBMCs) in the assessment of trastuzumab-induced cardiotoxicity remains uncertain. This study aimed to determine the temporal changes in mitochondrial function, oxidative stress, and cell death in the isolated PBMCs of HER2-positive breast cancer patients during breast cancer treatment and to compare the changes with HER2-negative breast cancer patients who did not receive trastuzumab therapy. Eighteen newly diagnosed HER2-positive breast cancer women who received sequential doxorubicin and trastuzumab were consecutively recruited. Age- and gender-matched controls with HER2-negative breast cancer were selected. Echocardiography was carried out, and blood samples for the study of cardiac biomarkers and PBMCs were collected periodically during treatment. Only one patient in our cohort developed asymptomatic left ventricular dysfunction during trastuzumab treatment. However, trastuzumab following doxorubicin aggravated subclinical cardiac injury, determined by cardiac troponin and echocardiography. Cellular and mitochondrial oxidative stress in isolated PBMCs remained unchanged throughout breast cancer treatment. Regarding mitochondrial respiration, the maximal respiration and spare respiration capacity was significantly increased in controls after doxorubicin treatment but not in patients who received trastuzumab therapy. Moreover, the percentage of apoptosis and necroptosis in isolated PBMCs was dramatically decreased in the control, compared to patients with trastuzumab treatment. In conclusion, trastuzumab caused subtle myocardial injury and impaired mitochondrial respiration and cell viability in isolated PBMCs.

## 1. Introduction

Trastuzumab, the first monoclonal antibody against human epidermal growth factor receptor 2 (HER2), is one of the cornerstones of HER2-positive breast cancer treatment. Immunotherapy with trastuzumab, in combination with chemotherapy, improves disease-free survival in patients with early-stage HER2-positive breast cancer [1,2,3] and increases time to disease progression in those with metastatic-stage cancer [4,5]. However, cardiotoxicity can unexpectedly develop and limits trastuzumab’s tolerability and efficacy in cancer treatment. Interestingly, the concomitant treatment of anthracycline and trastuzumab led to cardiac dysfunction in roughly one-quarter of patients, while an anthracycline-free regimen or sequential treatment could noticeably reduce the cardiac adverse effects of trastuzumab in 5–13% of patients [1,4]. These clinical findings demonstrate that anthracycline augments the cardiotoxicity of trastuzumab.

Trastuzumab-induced cardiotoxicity is possibly mediated through mitochondrial dysfunction and oxidative stress. From in vitro and in vivo studies, the inhibition of HER2 in cardiomyocytes deteriorated mitochondrial function and energy production [6,7,8]. Moreover, in combination with anthracycline, trastuzumab increased oxidative stress, leading to apoptosis [9]. However, no myocardial ultrastructure changes were reported in patients with clinical trastuzumab-induced cardiotoxicity, and cardiac dysfunction was generally reversible after trastuzumab interruption or discontinuation [10]. Currently, addressing the mechanism related to cardiotoxicity is still a challenging issue in research.

Peripheral blood mononuclear cells (PBMCs), which could be isolated from peripheral blood, consist of blood cells with single round nuclei, i.e., monocytes, lymphocytes, natural killer cells, and dendritic cells. The impairment of PBMC mitochondrial respiration has been reported in patients with heart failure [11,12,13]. Interestingly, mitochondrial dysfunction could be observed in asymptomatic individuals [11] and was related to the severity of heart failure [12]. Unfortunately, PBMCs are also involved in trastuzumab-mediated immunotherapy, called antibody-dependent cell-mediated cytotoxicity (ADCC) and phagocytosis [14]. The role of isolated PBMCs in the assessment of trastuzumab-induced cardiotoxicity, in terms of mechanistic evaluation and clinical detection, remains uncertain.

This study aimed to determine the temporal changes in mitochondrial function, oxidative stress, and cell death in isolated PBMCs throughout the clinical course of HER2-positive breast cancer patients receiving sequential doxorubicin and trastuzumab treatment. Moreover, we also compared the changes with HER2-negative breast cancer patients who received only doxorubicin-based chemotherapy. We hypothesized that trastuzumab impairs mitochondrial function and oxidative stress in isolated PBMCs.

## 2. Materials and Methods

### 2.1. Study Design

This study was a single-center, prospective, non-interventional cohort study conducted at Maharaj Nakorn Chiang Mai Hospital, Chiang Mai University, Thailand. The Ethics Committee of Faculty of Medicine, Chiang Mai University, approved the protocol of this study (Study Code: MED-2562-06859) on 10 August 2020. All eligible participants were recruited consecutively between September 2020 and August 2022, and written informed consent was obtained. The investigators reviewed and implemented this study, analyzed the study data, and prepared the manuscript for publication. The study reports were structured according to the Strengthening the Reporting of Observational studies in Epidemiology (STROBE) guidelines [15].

### 2.2. Patient Population

The observation cohort included women aged ≥18 years with newly diagnosed HER2-positive breast cancer. The treatment regimen consisted of adjuvant or neoadjuvant chemotherapy with 4 cycles of doxorubicin (60 mg/m^2^) and cyclophosphamide (600 mg/m^2^), followed by either intravenous or subcutaneous trastuzumab every 3 weeks. For the control cohort, age- and gender-matched patients with newly diagnosed HER2-negative breast cancer were recruited. Those without HER2 amplification received only adjuvant or neoadjuvant doxorubicin-based chemotherapy. Paclitaxel (175 mg/m^2^) every 3 weeks for 4 cycles was indicated postoperatively in patients with positive lymph node(s). Adjuvant hormone therapy was used according to the hormone receptor status, while adjuvant radiotherapy was considered according to clinicopathologic factors, including HER2 status. All recruited patients had apparently normal cardiac function at baseline, defined as left ventricular ejection fraction (LVEF) ≥ 53% prior to chemotherapy and radiotherapy. The exclusion criteria were advanced breast cancer at diagnosis, a prior history of other malignancies, chemotherapy or chest wall radiation, severe valvular heart disease, terminal illness (e.g., end-stage renal disease, cirrhosis) or life expectancy < 1 year, corrected QT interval ≥ 500 ms at baseline, and pregnancy or lactation. All participants received the standard of care according to comorbidities, provided by their primary physicians.

### 2.3. Data Collection

For the observation cohort, clinical, laboratory, and echocardiographic data were obtained at baseline (prior to chemotherapy and radiotherapy), after cycle 4 of doxorubicin-based chemotherapy, then every 4 cycles during trastuzumab treatment (month 3, 6, and 9), and >1 month after the completion of trastuzumab treatment. The follow-up visit at month 9 during trastuzumab treatment was omitted if the patients received trastuzumab <9 months. To compare with patients who did not receive trastuzumab, the data were collected at baseline, after cycle 4 of doxorubicin-based chemotherapy, and at 1 year from baseline (comparable to month 9 of trastuzumab treatment) in the control cohort. Blood samples for laboratory tests included high-sensitivity cardiac troponin I (hs-cTnI), N-terminal pro B-type natriuretic peptide (NT-proBNP), creatine kinase MB isoenzyme (CK-MB), and the analysis of isolated PBMCs for mitochondrial respiration, oxidative stress, and cell death.

### 2.4. Cardiac Biomarkers

Chemiluminescent microparticle immunoassay was used for hs-cTnI using the Abbott/Architect stat hsTnI assay ARCHITECT i2000SR Diagnostic System (Abbott Laboratories, Wiesbaden, Germany). The 99th percentile concentration of hs-cTnI for female patients was 17 ng/L with a coefficient of variation of 5.0%. For patients with hs-cTnI <2.0 ng/L, the limit of detection, we decided to use 2.0 ng/L for the data analysis. NT-proBNP was measured by electrochemiluminescence immunoassay (ECLIA) using the Cobas e601 system (Roche Diagnostics, Mannheim, Germany). The measuring range of NT-proBNP was 10–35,000 pg/mL. NT-proBNP ≥125 pg/mL was considered as a significant rise beyond biological variation [16]. Lastly, CK-MB was determined by ECLIA using the Cobas 8000 system (Roche Diagnostics, Mannheim, Germany).

### 2.5. The Analysis of Mitochondrial Respiration, Oxidative Stress, and Cell Death in PBMCs

A total of 12 mL of venous blood was obtained for PBMC isolation, using a Ficoll density gradient centrifugation. After initial centrifugation at 1000× *g* for 10 min, the plasma was discarded. Buffy coat and red blood cells were re-suspended in phosphate buffer saline (PBS) solution, then carefully layered onto a Histopaque reagent (Sigma-Aldrich, St. Louis, MO, USA), and centrifuged at 400× *g* for 30 min. The ring of PBMCs was harvested and washed twice with PBS solution. After final centrifugation at 1000× *g* for 10 min, cell counting was performed using a hemocytometer. Mitochondrial function was determined using a mitochondrial stress test kit, and the oxygen consumption rate was measured using a high-throughput automated 96-well extracellular flux analyzer (XFe96; Agilent Seahorse, Santa Clara, CA, USA) and automatically analyzed by an analytical program (Wave; Agilent Seahorse, USA). Mitochondrial reactive oxygen species (ROS) levels were measured using MitoSOX Red Staining (Thermo Fisher, Waltham, MA, USA), while MitoTracker green Staining (Thermo Fisher, Waltham, MA, USA) was used to determine mitochondrial mass in isolated PBMCs. The staining fluorescent intensity was measured by flow cytometry (FACS Celesta, BD biosciences, Franklin Lakes, NJ, USA). Moreover, to quantify the percentage of apoptotic cells, 2 × 10^5^ cells were stained with the FITC Annexin V apoptosis detection kit (BD biosciences, Franklin Lakes, NJ, USA), and the propidium iodide flow cytometry assay was used to access cell viability.

### 2.6. Echocardiography

Transthoracic echocardiography was performed using the EPIQ CVx ultrasound system (Koninklijke Philips N.V., Amsterdam, The Netherlands), equipped with an X5-1 or X5-1c transducer (frequency range 5–1 MHz). The imaging data were exported to IntelliSpace Cardiovascular (Koninklijke Philips N.V., The Netherlands) for off-cart measurement using an echo module. LVEF was measured by 2D modified Simpson’s method, and left ventricular global longitudinal strain (GLS) by 2D speckle-tracking echocardiography was calculated using AutoStrain LV (TOMTEC, Unterschleißheim, Germany). Cardiac chamber quantification was measured according to the recommendations of the American Society of Echocardiography and the European Association of Cardiovascular Imaging [17]. Cancer therapy-related cardiac dysfunction (CTRCD) was defined as new LVEF reduction ≥ 10% to the value < 53%, regardless of symptoms [18]. Symptomatic CTRCD represented heart failure, consisting of symptoms (e.g., dyspnea on exertion, orthopnea, fatigue, ankle swelling) and/or signs (e.g., elevated jugular venous pressure, rales, dependent edema) [16]. A >15% relative reduction in GLS was considered as subclinical left ventricular dysfunction [19].

### 2.7. Statistical Analysis

The categorical data were presented as numbers (percentages), while the continuous data were presented as the mean ± standard deviation in a normal distribution or median (interquartile range) in a non-normal distribution. A one-way repeated measures ANOVA was used to determine the significance of temporal changes in study parameters within the cohorts. Then, Tukey’s multiple comparison test was subsequently performed to identify a different pair, where appropriate. HER2-positive breast cancer patients who received postoperative trastuzumab ≥ 9 months in the observation cohort were selected for a case-control comparison with 1:1 matched patients with HER2-negative breast cancer in the control cohort. Between both cohorts, the categorical data were compared using Fisher’s exact test, and the Wilcoxon test for matched samples was used for continuous data. For bar graphs, data were presented as the mean with T bars indicating standard error. As a result of a small number of missing data, multiple imputation was not used. The *p*-value for a 2-sided test of <0.05 was considered as statistical significance. The statistical analyses were conducted with the Stata 16 software (StataCorp LLC, College Station, TX, USA) and Prism 10 (GraphPad Software, LLC, Boston, MA, USA).

## 3. Results

### 3.1. Patients

**Figure 1:**
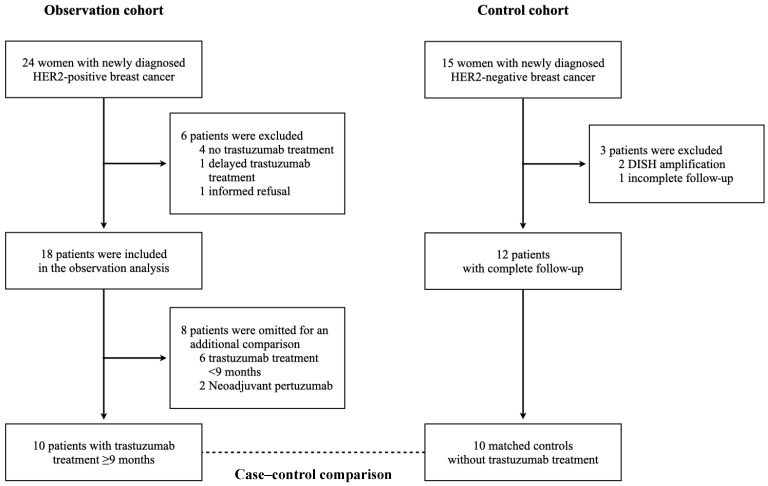
Selection criteria for data analysis. STROBE flow chart demonstrates eligible patients in observation cohort and control cohort. DISH, dual in situ hybridization; HER2, human epidermal growth factor receptor 2.

From September 2020 to August 2022, 24 women with newly diagnosed HER2-positive breast cancer were potentially eligible, and a total of 18 patients were included in the observation cohort (Figure 1). The mean age of patients at the time of breast cancer diagnosis was 48.8 ± 9.1 years. Most patients had no previous cardiovascular risk factors. Specifically, no patients had a history of smoking, diabetes, or atherosclerotic cardiovascular disease. Prior to breast cancer treatment, one-third of patients already used cardioprotective drugs, either angiotensin-converting enzyme inhibitors (ACEIs), angiotensin receptor blockers (ARBs), beta-blockers (BBs), or statins, for their cardiovascular conditions. The average total dose of doxorubicin was 223 ± 8 mg/m^2^, and one-third of patients were prescribed as a neoadjuvant treatment. All patients underwent breast cancer surgery, and additional locoregional treatment with radiotherapy was prescribed postoperatively in most cases. Two-thirds of patients received trastuzumab every 3 weeks for 18 cycles. Neoadjuvant trastuzumab in combination with pertuzumab for 4 cycles, then postoperative trastuzumab for 14 cycles, was observed in two participants (Table 1).

### 3.2. Clinical Data, Cardiac Biomarkers, and Echocardiography

**Figure 2:**
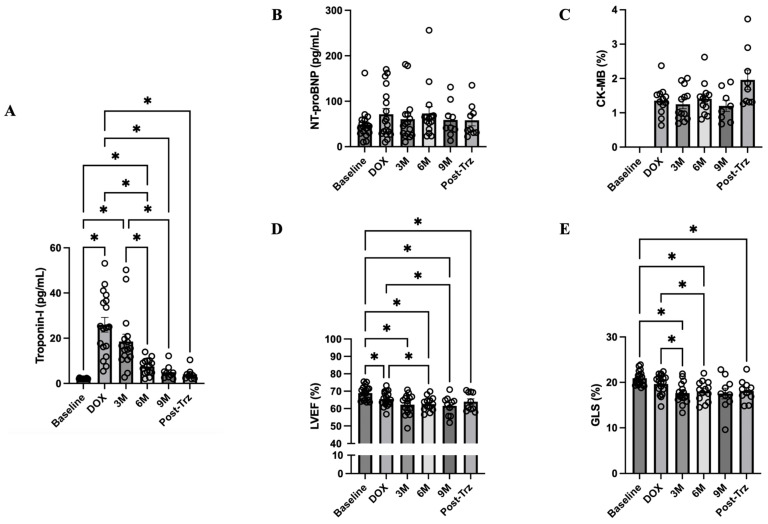
Cardiac biomarkers and left ventricular function in patients with HER2-positive breast cancer. (**A**) Serum concentration of hs-cTnI. (**B**) Serum concentration of NT-proBNP. (**C**) Serum activity of CK-MB. (**D**,**E**) LVEF and GLS measured by echocardiography. Bar graphs represent data at different timepoints during treatment: Baseline = prior to all chemotherapy and radiotherapy, DOX = after completion of doxorubicin-based chemotherapy, 3M = month 3 during trastuzumab treatment, 6M = month 6 during trastuzumab treatment, 9M = month 9 during trastuzumab treatment, and Post-Trz = after completion of trastuzumab treatment > 1 month. CK-MB data at baseline were not available. * *p* < 0.05. CK-MB, creatine kinase MB isoenzyme; GLS, global longitudinal strain; HER2, human epidermal growth factor receptor 2; hs-cTnI, high-sensitivity cardiac troponin I; LVEF, left ventricular ejection fraction; NT-proBNP, N-terminal pro B-type natriuretic peptide.

After doxorubicin treatment, hs-cTnI concentrations were significantly increased, then gradually decreased afterward during trastuzumab treatment (Figure 2A). Two-thirds of patients had elevated hs-cTnI > 99th percentile concentration after the completion of four cycles of doxorubicin-based chemotherapy. Interestingly, the concentrations of hs-cTnI were completely normal at month 6 during trastuzumab treatment. NT-proBNP concentrations and CK-MB activities remained statistically unchanged throughout the course of treatment (Figure 2B,C). A reduction in LVEF and GLS was demonstrated after doxorubicin treatment, and an additional reduction was observed during trastuzumab treatment (Figure 2D,E). A >15% relative reduction in GLS was observed in 3 of 18 patients (16.7%) after doxorubicin treatment. Moreover, the number of patients with subclinical left ventricular dysfunction increased to 11 of 18 (61.1%) during trastuzumab treatment. In the observation cohort, only one patient developed CTRCD during trastuzumab treatment. This patient did not have any symptoms or signs of heart failure. After withholding trastuzumab dosing for 6 weeks, together with prescribing ACEI and BB, LVEF was partially improved, and 12-month trastuzumab treatment could be completed subsequently.

### 3.3. Mitochondrial Respiration, Oxidative Stress, and Cell Death in PBMCs

**Figure 3:**
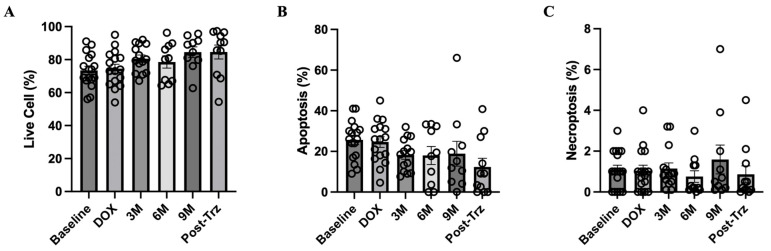
PBMC analysis of live cells, apoptosis, and necroptosis in patients with HER2-positive breast cancer. (**A**) Percentage of live cells. (**B**) Percentage of apoptosis. (**C**) Percentage of necroptosis. Bar graphs represent data at different timepoints during treatment: Baseline = prior to all chemotherapy and radiotherapy, DOX = after completion of doxorubicin-based chemotherapy, 3M = month 3 during trastuzumab treatment, 6M = month 6 during trastuzumab treatment, 9M = month 9 during trastuzumab treatment, and Post-Trz = after completion of trastuzumab treatment > 1 month. HER2, human epidermal growth factor receptor 2; PBMC, peripheral blood mononuclear cell.

Mitochondrial respiration and oxidative stress in isolated PBMCs were unchanged during doxorubicin-based chemotherapy and trastuzumab treatment (Figures S1 and S2 in the Supplementary Materials). The percentage of live cells in PBMCs tended to increase during the treatment (Figure 3A). While the percentage of apoptosis tended to decrease over time, the percentage of necroptosis was unchanged (Figure 3B,C).

### 3.4. Case-Control Comparison

**Figure 4:**
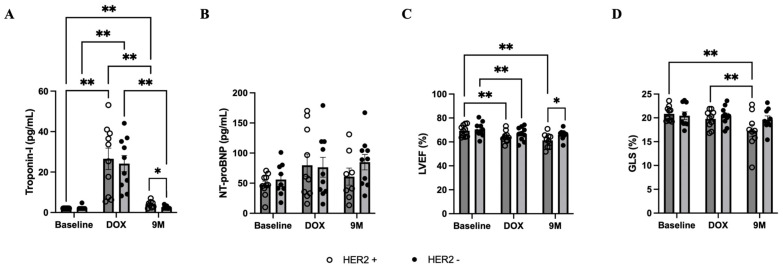
Cardiac biomarkers and left ventricular function during treatment, showing comparisons between groups. (**A**) Serum concentration of hs-cTnI. (**B**) Serum concentration of NT-proBNP. (**C**,**D**) LVEF and GLS measured by echocardiography. Bar graphs represent data at different timepoints during treatment: Baseline = prior to all chemotherapy and radiotherapy, DOX = after completion of doxorubicin-based chemotherapy, and 9M = month 9 during trastuzumab treatment. * *p* < 0.05 between groups, and ** *p* < 0.05 within group. GLS, global longitudinal strain; HER2, human epidermal growth factor receptor 2; hs-cTnI, high-sensitivity cardiac troponin I; LVEF, left ventricular ejection fraction; NT-proBNP, N-terminal pro B-type natriuretic peptide.

There were 10 HER2-positive breast cancer patients who received postoperative trastuzumab treatment ≥ 9 months in the observation cohort. To determine the effects of trastuzumab treatment, 10 age- and gender-matched controls without trastuzumab treatment were selected from the control cohort (Figure 1). Baseline characteristics were similar between groups; however, the mean age of patients with HER2-negative breast cancer was numerically higher than those with HER2-positive breast cancer (Table 1). At month 9 after doxorubicin treatment, the concentrations of hs-cTnI were normalized in both groups, but significantly lower concentrations were observed in patients without trastuzumab treatment (4.1 vs. 2.5 ng/L, *p* = 0.02) (Figure 4A). NT-proBNP concentrations were similar in both groups throughout the course of treatment (Figure 4B). During trastuzumab treatment, LVEF and GLS were additionally reduced, and the mean LVEF was significantly lower in patients with HER2-positive breast cancer (61.3 vs. 66.0%, *p* = 0.04) (Figure 4C,D).

**Figure 5:**
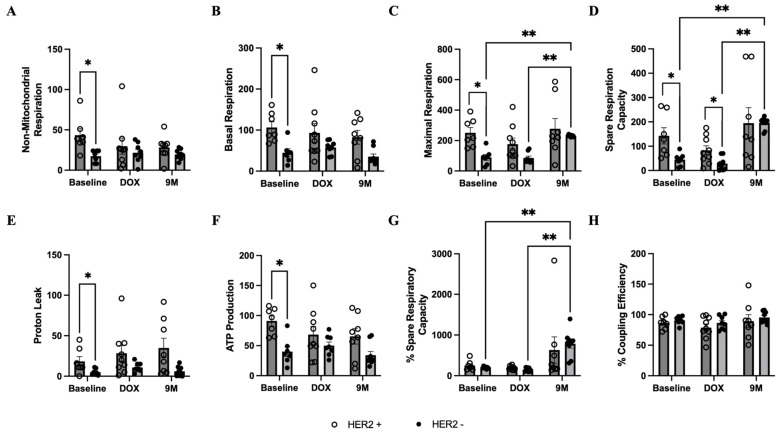
Mitochondrial respiration in isolated PBMCs during treatment, showing comparisons between groups. Oxygen consumption rate (pmol/min) measured by Agilent Seahorse test is demonstrated in graph (**A**) non-mitochondrial respiration, (**B**) basal respiration, (**C**) maximal respiration, (**D**) spare respiration capacity, (**E**) proton leak, and (**F**) ATP production. (**G**) Percentage of spare respiratory capacity. (**H**) Percentage of coupling efficiency. Bar graphs represent data at different timepoints during treatment: Baseline = prior to all chemotherapy and radiotherapy, DOX = after completion of doxorubicin-based chemotherapy, and 9M = month 9 during trastuzumab treatment. * *p* < 0.05 between groups, and ** *p* < 0.05 within group. ATP, adenosine triphosphate; HER2, human epidermal growth factor receptor 2; PBMC, peripheral blood mononuclear cell.

**Figure 6:**
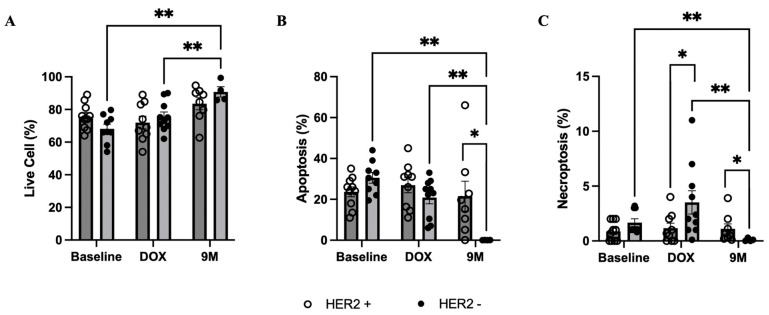
PBMC analysis of live cells, apoptosis, and necroptosis during treatment, showing comparisons between groups. (**A**) Percentage of live cells. (**B**) Percentage of apoptosis. (**C**) Percentage of necroptosis. Bar graphs represent data at different timepoints during treatment: Baseline = prior to all chemotherapy and radiotherapy, DOX = after completion of doxorubicin-based chemotherapy, and 9M = month 9 during trastuzumab treatment. * *p* < 0.05 between groups, and ** *p* < 0.05 within group. HER2, human epidermal growth factor receptor 2; PBMC, peripheral blood mononuclear cell.

Patients with HER2-negative breast cancer had lower non-mitochondrial and mitochondrial respiration, including proton leak and adenosine triphosphate (ATP) production, in isolated PBMCs at baseline, comparing to those with HER2-positive breast cancer (Figure 5A–F). However, the percentage of spare respiratory capacity and coupling efficiency was similar between groups (Figure 5G,H). At month 9 after doxorubicin treatment, the maximal respiration and spare respiration capacity was significantly increased in patients without trastuzumab treatment, while a non-significant increase was also observed in those with trastuzumab treatment. Oxidative stress in isolated PBMCs was unchanged in both groups during treatment (Figure S3). In patients with HER2-negative breast cancer, the percentage of live cells in PBMCs was significantly increased, while the percentage of apoptosis and necroptosis decreased at month 9 after chemotherapy completion (Figure 6A–C). The percentage of apoptosis and necroptosis in PBMCs was significantly higher in patients who received trastuzumab treatment (Figure 6B,C).

## 4. Discussion

This is the first study demonstrating the temporal changes in mitochondrial respiration and oxidative stress in isolated PBMCs throughout the course of breast cancer treatment, specifically from pre-treatment, post-doxorubicin treatment, during trastuzumab treatment, and until treatment completion. Moreover, we compared the changes with controls who did not receive trastuzumab treatment.

A prior study in patients receiving epirubicin-based chemotherapy showed that mitochondrial respiration in isolated PBMCs was increased in average 3 months after adjuvant chemotherapy with or without radiation [20]. In another study, the maximum oxygen consumption rate was increased over time after neoadjuvant chemotherapy completion in breast cancer patients [21]. Similar findings were found in our study. The maximum respiration and spare respiration capacity in patients without trastuzumab treatment was significantly increased at month 9 after doxorubicin treatment. Interestingly, treating with trastuzumab limited the increase. This finding suggests that trastuzumab might impair the metabolic adaptation of mitochondria in PBMCs.

After doxorubicin treatment, the concentrations of hs-cTnI were gradually decreased regardless of trastuzumab treatment. This finding supports that trastuzumab does not cause direct injury to the myocardium, as previously described [18]. However, hs-cTnI in patients without trastuzumab treatment was significantly lower, compared to those with trastuzumab treatment. Moreover, LVEF reduction and subclinical left ventricular dysfunction, determined by GLS, were continuously detected in patients receiving trastuzumab treatment, while no additional left ventricular dysfunction was observed after chemotherapy completion in patients without trastuzumab treatment. These findings indicate that trastuzumab causes subtle injury to myocardium. However, the left ventricular function after trastuzumab treatment was comparable to its level after doxorubicin treatment. The temporal changes in left ventricular function were similar to the previous report [22]. This recovery might indicate the reversibility of trastuzumab-induced cardiotoxicity after treatment. In both cohorts, NT-proBNP was unchanged during and after treatment. Only one patient developed asymptomatic CTRCD in our study. Although NT-proBNP is a clinical marker of increased left ventricular filling pressure, it might not be sensitive enough to determine subtle myocardial injury.

A prior report demonstrated increased oxidative stress in isolated PBMCs in patients with gastrointestinal cancer, compared to healthy individuals [23]. Our study showed that both cellular and mitochondrial oxidative stress in isolated PBMCs was unchanged during breast cancer treatment, regardless of trastuzumab treatment. The oxidative stress in PBMCs was associated with the severity of heart failure [12]; however, it might not represent the level of oxidative stress in myocardial cells, especially in asymptomatic patients.

In patients without trastuzumab treatment, the percentage of live cells in PBMCs was significantly increased, while the percentage of apoptosis and necroptosis was markedly reduced at month 9 after doxorubicin treatment. Interestingly, in HER2-positive breast cancer patients, the percentage of apoptosis and necroptosis was persistently elevated during trastuzumab treatment. ADCC might be involved in this phenomenon. In the neoadjuvant trial ICORG10-05, HER2-positive breast cancer patients were treated with chemotherapy in combination with trastuzumab, lapatinib, or both [24]. The treatment altered the differentiation of PBMCs toward an increase in the percentage of T cells.

There were several limitations in this study. First, the number of observed patients was small, despite the complete course of breast cancer treatment and few missing data. Second, the cumulative dose of doxorubicin was relatively low. The higher total dose of anthracyclines might result in the alteration of clinical findings, as well as mitochondrial respiration and oxidative stress in isolated PBMCs. Third, even though cases with trastuzumab treatment and controls were well matched, non-mitochondrial and mitochondrial respiration in PBMCs at baseline were significantly higher in patients with HER2-negative breast cancer, compared to those with HER2-positive breast cancer. This could possibly be the result of a statistically non-significant difference in age, other undetermined factors or comorbidities, or somatic mutation in HER2. Lastly, only one patient developed CTRCD without any symptoms and signs of heart failure.

## 5. Conclusions

Trastuzumab caused subtle myocardial injuries. The delay of hs-cTnI resolution and additional left ventricular dysfunction was observed during trastuzumab treatment after doxorubicin chemotherapy. Moreover, trastuzumab treatment affected the improvement in the maximum respiration and spare respiration capacity of mitochondria and cell viability in isolated PBMCs. However, both cellular and mitochondrial oxidative stress in isolated PBMCs was unchanged during breast cancer treatment.

## Figures and Tables

**Table 1:** Baseline characteristics and clinical data of participants in observation cohort and control cohort.

Clinical Data	Observation Cohort	Case-Control Comparison		
	HER2 + (*n* = 18)	HER2 + (*n* = 10)	HER2 − (*n* = 10)	*p*-Value ^a^
Age (years)	48.8 ± 9.1	46.5 ± 10.7	49.0 ± 7.5	0.55
Height (cm)	157.4 ± 6.0	156.6 ± 6.4	156.2 ± 6.8	0.89
Weight (kg)	59.9 ± 9.8	57.7 ± 7.6	57.0 ± 14.6	0.90
Serum creatinine (mg/dL)	0.70 ± 0.15	0.67 ± 0.14	0.70 ± 0.13	0.55
Comorbidities (%)				
Hypertension	2 (11.1)	1 (10)	1 (10)	1.00
Dyslipidemia	6 (33.3)	2 (20)	2 (20)	1.00
Diabetes	0 (0)	0 (0)	0 (0)	-
Atherosclerotic CVD	0 (0)	0 (0)	0 (0)	-
Smoking history (%)	0 (0)	0 (0)	0 (0)	-
Left-side cancer (%)	8 (44.4)	4 (40)	5 (50)	1.00
Doxorubicin chemotherapy				
Total dose (mg/m^2^)	223 ± 8	224 ± 9	222 ± 10	0.72
Neoadjuvant setting (%)	6 (33.3)	4 (40)	3 (30)	1.00
Trastuzumab treatment (%)			N/A	-
Subcutaneous administration	9 (50)	3 (30)		
Neoadjuvant setting (combined with pertuzumab)	2 (11.1)	0 (0)		
≥9 months postoperative to treatment	12 (66.7)	10 (100)		
Radiotherapy (%)	16 (88.9)	10 (100)	5 (50)	0.03
Medications (%)				
ACEIs or ARBs	2 (11.1)	1 (10)	0 (0)	-
BBs	1 (5.6)	0 (0)	1 (10)	-
Statins	5 (27.8)	1 (10)	0 (0)	-

Abbreviations: ACEI, angiotensin-converting enzyme inhibitor; ARB, angiotensin receptor blocker; BB, beta-blocker; CVD, cardiovascular disease; HER2, human epidermal growth factor receptor 2; N/A, not available. ^a^ *p*-values represent the comparison between HER2-positive and HER2-negative patients.

## Data Availability

The original contributions presented in the study are included in the article/Supplementary Materials, further inquiries can be directed to the corresponding author.

## Supplementary Materials

The following supporting information can be downloaded at https://www.mdpi.com/article/10.3390/biomedicines12091970/s1. Figure S1. Mitochondrial respiration in isolated PBMCs during treatment in patients with HER2-positive breast cancer. Figure S2. Oxidative stress in isolated PBMCs during treatment in patients with HER2-positive breast cancer. Figure S3. Oxidative stress in isolated PBMCs during treatment between patients with HER2-positive breast cancer and patients with HER2-negative breast cancer.
