# Supplementary material for: Changes in Mitochondrial Function and Cell Death Patterns in Peripheral Blood Mononuclear Cells during Trastuzumab Treatment Following Doxorubicin Chemotherapy
Source: Biomedicines. 2024 Sep 1;12(9):1970. doi: 10.3390/biomedicines12091970 (PMC11429371; doi:10.3390/biomedicines12091970)
Supplement: Supplementary file 1 [file biomedicines-12-01970-s001.zip › biomedicines-3177865-supplementary.pdf]

Supplementary Material

Changes in Mitochondrial Function and Cell Death Patterns in Peripheral Blood Mononuclear Cells during Trastuzumab Treatment Following Doxorubicin Chemotherapy

Biomedicines

Krit Leemasawat [1,2,3], Nichanan Osataphan [1,2,3], Nattayaporn Apaijai [2,3], Panat Yanpiset [2,3], Arintaya Phrommintikul [1,2,3], Areewan Somwangprasert [4], Siriporn C. Chattipakorn [2,3,5] and Nipon Chattipakorn [2,3,6,*]

1 Cardiology Division, Department of Internal Medicine, Faculty of Medicine, Chiang Mai University, Chiang Mai 50200, Thailand
2 Cardiac Electrophysiology Research and Training Center, Faculty of Medicine, Chiang Mai University, Chiang Mai 50200, Thailand
3 Center of Excellence in Cardiac Electrophysiology Research, Chiang Mai University, Chiang Mai 50200, Thailand
4 Department of Surgery, Faculty of Medicine, Chiang Mai University, Chiang Mai 50200, Thailand
5 Department of Oral Biology and Diagnostic Sciences, Faculty of Dentistry, Chiang Mai University, Chiang Mai 50200, Thailand
6 Cardiac Electrophysiology Unit, Department of Physiology, Faculty of Medicine, Chiang Mai University, Chiang Mai 50200, Thailand
* Correspondence: nchattip@gmail.com

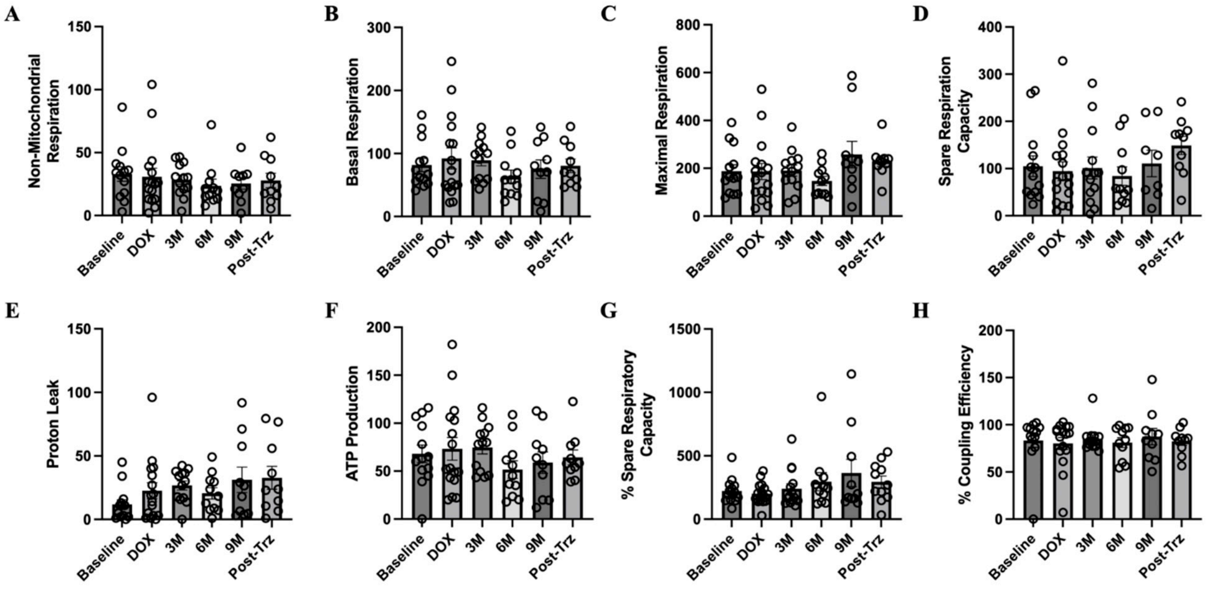

Figure S1. Mitochondrial respiration in isolated PBMCs during treatment in patients with HER2-positive breast cancer. Oxygen consumption rate (pmol/min) measured by Agilent Seahorse test is demonstrated in graph (A) non-mitochondrial respiration, (B) basal respiration, (C) maximal respiration, (D) spare respiration capacity, (E) proton leak, and (F) ATP production. (G) Percentage of spare respiratory capacity. (H) Percentage of coupling efficiency. Bar graphs represent the data at different timepoints during treatment: Baseline = Prior to all chemotherapy and radiotherapy, DOX = After completion of doxorubicin-based chemotherapy, 3M = Month 3 during trastuzumab treatment, 6M = Month 6 during trastuzumab treatment, 9M = Month 9 during trastuzumab treatment, and Post-Trz = After completion of trastuzumab treatment >1 month. ATP, Adenosine triphosphate; HER2, Human epidermal growth factor receptor 2; PBMC, Peripheral blood mononuclear cell.

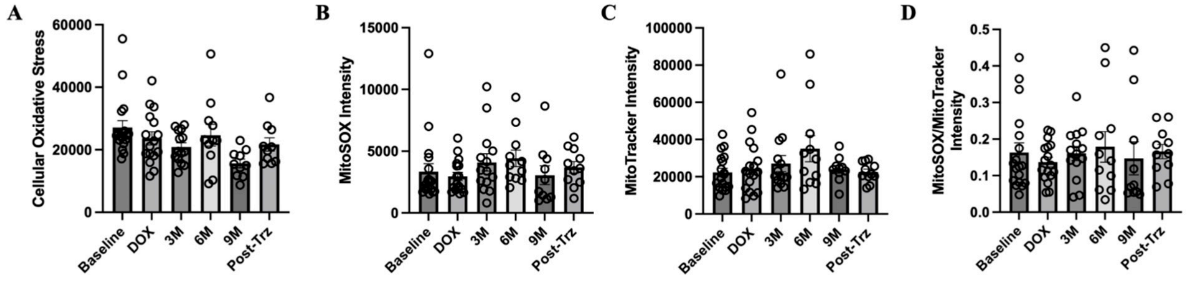

Figure S2. Oxidative stress in isolated PBMCs during treatment in patients with HER2-positive breast cancer. (A) Cellular oxidative stress. (B) MitoSOX. (C) MitoTracker. (D) MitoSOX/MitoTracker. Data are presented as the mean fluorescence intensity. Bar graphs represent the data at different timepoints during treatment: Baseline = Prior to all chemotherapy and radiotherapy, DOX = After completion of doxorubicin-based chemotherapy, 3M = Month 3 during trastuzumab treatment, 6M = Month 6 during trastuzumab treatment, 9M = Month 9 during trastuzumab treatment, and Post-Trz = After completion of trastuzumab treatment >1 month. HER2, Human epidermal growth factor receptor 2; PBMC, Peripheral blood mononuclear cell.

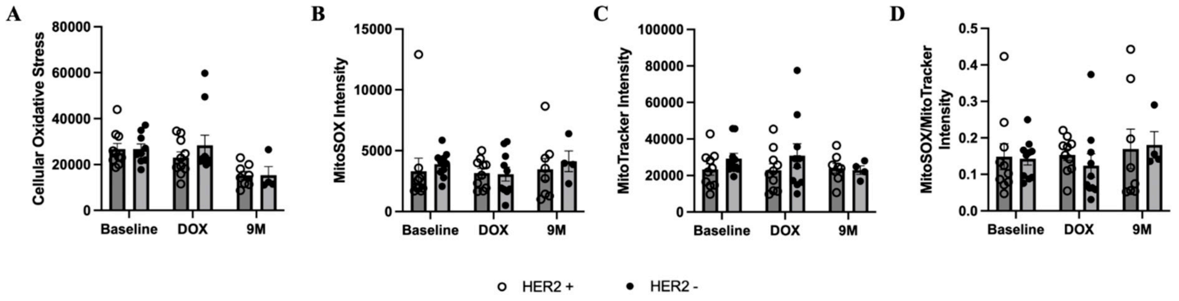

Figure S3. Oxidative stress in isolated PBMCs during treatment between patients with HER2-positive breast cancer and patients with HER2-negative breast cancer. (A) Cellular oxidative stress. (B) MitoSOX. (C) MitoTracker. (D) MitoSOX/MitoTracker. Data are presented as the mean fluorescence intensity. Bar graphs represent the data at different timepoints during treatment: Baseline = Prior to all chemotherapy and radiotherapy, DOX = After completion of doxorubicin-based chemotherapy, and 9M = Month 9 during trastuzumab treatment. HER2, Human epidermal growth factor receptor 2; PBMC, Peripheral blood mononuclear cell.
